# Simulating Absorption Spectra of Flavonoids in Aqueous Solution: A Polarizable QM/MM Study

**DOI:** 10.3390/molecules25245853

**Published:** 2020-12-11

**Authors:** Sulejman Skoko, Matteo Ambrosetti, Tommaso Giovannini, Chiara Cappelli

**Affiliations:** 1 Scuola Normale Superiore, Piazza dei Cavalieri 7, I-56126 Pisa, Italy; sulejman.skoko@sns.it (S.S.); matteo.ambrosetti@sns.it (M.A.); 2 Department of Chemistry, Norwegian University of Science and Technology (NTNU), 7491 Trondheim, Norway; tommaso.giovannini@ntnu.no

**Keywords:** flavonoids, QM/MM, MD, QM/FQ, absorption spectrum, UV/Vis

## Abstract

We present a detailed computational study of the UV/Vis spectra of four relevant flavonoids in aqueous solution, namely luteolin, kaempferol, quercetin, and myricetin. The absorption spectra are simulated by exploiting a fully polarizable quantum mechanical (QM)/molecular mechanics (MM) model, based on the fluctuating charge (FQ) force field. Such a model is coupled with configurational sampling obtained by performing classical molecular dynamics (MD) simulations. The calculated QM/FQ spectra are compared with the experiments. We show that an accurate reproduction of the UV/Vis spectra of the selected flavonoids can be obtained by appropriately taking into account the role of configurational sampling, polarization, and hydrogen bonding interactions.

## 1. Introduction

Flavonoids belong to the family of polyphenolic secondary metabolites, which are widely found in natural products, such as vegetables and fruits [1]. In particular, their structure derives from phenylchromene, which after being hydroxylated or methoxylated in different positions leads to the different flavonoid compounds [2]. Flavonoids have attracted much interest, due to their biochemical and antioxidant effects which might be beneficial to treat different diseases such as cancer, Alzheimer’s disease, and atherosclerosis [3,4,5]. Thanks to their antioxidant and anti-inflammatory properties, together with their ability to inhibit enzyme functions, a large number of flavonoid compounds have been used in a plethora of medicinal, pharmaceutical, and cosmetic applications [6,7,8]. Recently, flavonoids have been proposed as potential drugs for therapeutics against coronavirus disease 2019 (COVID-19) [9,10,11].

In addition to their therapeutic usages, flavonoids play a crucial biological activities in plants and animals [12,13,14,15,16]. In plants, flavonoids are responsible for the colour and aroma of flowers and fruits [17,18,19,20]. One of the most relevant examples is given by wine, and in particular red wine, in which flavonoids provide organoleptic and disease prevention properties [21,22,23,24,25,26].

**Figure 1:**
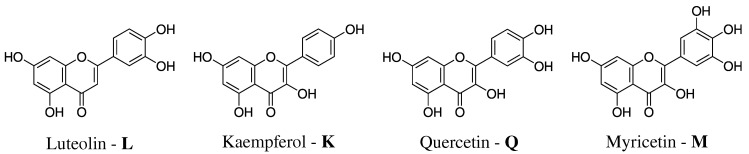
Molecular structure of the studied Flavonoids.

Flavonoids also protect plants thanks to their ability to filter the UV radiation, indicating unique optical properties [27]. The latter are very sensitive to the environment, for instance flavonoids’ maximum UV/Vis absorption occurs at different wavelengths when they are dissolved in different solvents. However, flavonoids natural environment is water, which is ubiquitous in both vegetables and fruits [28,29,30]. In this work, we model the absorption spectra of Luteolin (**L**), Kaempferol (**K**), Quercetin (**Q**), and Myricetin (**M**) in aqueous solution (see Figure 1 for their molecular structures) [28]. Luteolin is a yellow dye, which can be derived from the plant Reseda luteola [31]; kaempferol can be found in different vegetables [32], whereas quercetin and myricetin are among the most diffuse flavonoids in red wine [24,33,34]. As it can be noticed from Figure 1, all selected flavonoids are characterized by several OH groups, which can be involved in Hydrogen Bonding (HB) interactions with the surrounding aqueous environment.

From the theoretical point of view, in order to accurately simulate the absorption spectra of dyes in aqueous solution, both the solute and the solvent molecules have to be described atomistically to capture specific solute–solvent interactions, such as HB [35,36,37,38]. These kinds of problems are usually treated by resorting to the so-called focused models [35,39,40,41,42], which are based on the assumption that the solute gives rise to the spectral property, which is modified, but not determined, by the external environment. Within this framework, the solute is treated at the Quantum Mechanical (QM) level, whereas the solvent is described classically either by exploiting an implicit continuum approach [40,43], or an explicit atomistic model. When specific (HB) interactions take place between the solute and the solvent, the atomistic description of the environment usually overperforms the implicit continuum approach [37,44,45,46,47]. In most atomistic approaches, the solvent is described by means of a classical force field (FF), resulting in the QM/Molecular Mechanics (QM/MM) methods, in which the interaction between the QM and MM portions is described in terms of classical electrostatics [48,49,50,51]. The mutual polarization between the two portions can be introduced by resorting to polarizable QM/MM methods [52,53,54,55,56,57,58].

In this work, the absorption spectra of the selected flavonoids in aqueous solution (see Figure 1) are calculated by exploiting the polarizable QM/Fluctuating Charge (QM/FQ) approach [35,36,42]. In this scheme, the solute is treated at the QM level, and the solvent water molecules are described by means of the FQ force field [59,60,61]. In particular, each MM atom is endowed with a charge, which can fluctuate as a response to the QM potential [54]. The FQs enter in the definition of the QM Hamiltonian and the Self Consistent Field procedure is iterated until a mutual solute–solvent polarization is reached [54]. The QM/FQ method is usually coupled with classical Molecular Dynamics (MD), which allows for taking into account the dynamical aspect of the solvation phenomenon [46,62,63,64]. Such an approach has been shown to be particularly suitable to accurately describe the properties of aqueous solutions [35,36]. Major details on its approach can be found in a recent review by some of the present authors [35].

The manuscript is organized as follows. In the next section, we discuss the computational protocol which is exploited in the calculations. The numerical results and a discussion of the main findings of the present study are then presented. A summary and some conclusions end the manuscript.

## 2. Results and Discussion

In this section, we first discuss the computational protocol exploited in the calculations. Then, MD results for each flavonoid depicted in Figure 1 are presented, by analyzing conformational distributions of the main dihedral angles and hydrogen bonding patterns. Then, QM/FQ absorption spectra are presented and compared to experiments.

### 2.1. Computational Protocol

**Figure 2:**
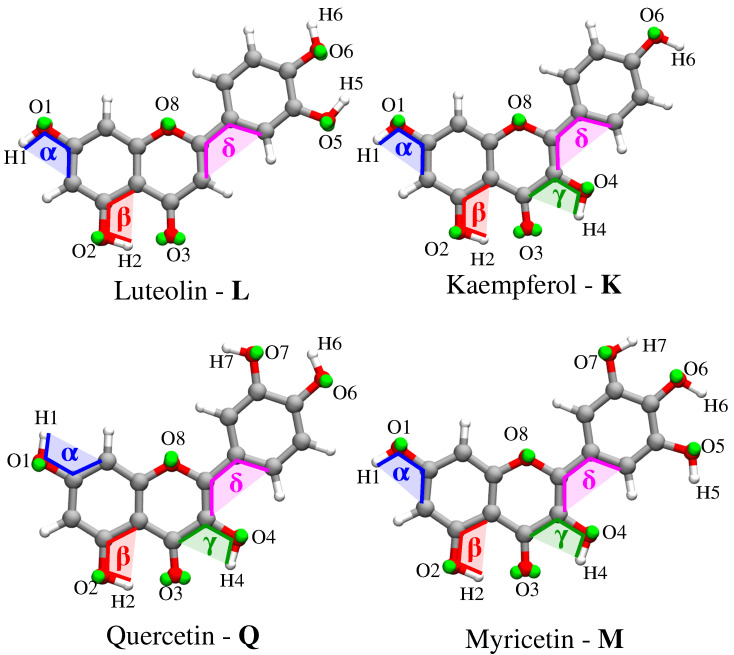
Atom labeling and definition of dihedral angles and oxygen virtual sites for the four flavonoids.

In order to investigate the electronic properties of the selected molecules dissolved in aqueous solution, we rely on a well established multi-step computational protocol [35,36]:

1. Definition of the system. The geometry of each of the four flavonoids depicted in Figure 1 was first optimized by resorting to an implicit Polarizable Continuum Model (PCM) [40] description of the solvent, and then surrounded by a number of randomly-placed water molecules large enough to represent the solvation shell.
2. Classical MD simulations. An equilibration (NPT) and a subsequent production (NVT) runs were performed in order to sample the system under study. In particular, MD production runs were carried out for each of the four molecules for a time long enough to obtain an accurate sampling of the phase space, in order to correctly reproduce all possible system configurations and their relative energy.
  In order to recover the directionality of hydrogen bonding (HB) interactions, we also placed off-site charges (the so-called virtual sites (VS) or dummy atoms) to better describe the lone pairs of the Oxygen atom (see Figure 2 for a graphical representation) [44,65,66]. Therefore, two different classical MD simulation runs were performed for each molecule, i.e., with or without the inclusion of VS (MD${}_{\mathrm{VS}}$ or MD${}_{\mathrm{noVS}}$, respectively). From MD runs, a set of snapshots was extracted to be used in QM/FQ calculations.
3. Definition of the different regions of the two-layer QM/FQ scheme and their boundaries. For each snapshot extracted from MD runs, a sphere centered on the solute was cut. The radius of the droplet was chosen to retain specific solute–water interactions.
4. QM/FQ calculations and comparison with experimental data reported in [28,67]. QM/FQ excitation energies calculations were performed on the set of structures obtained for the four molecules at step 2 of the protocol. The results obtained for each spherical snapshot were then extracted and averaged to produce the final spectrum, which was compared with experiments.

All QM calculations were performed using a locally modified version of the Gaussian16 suite [68]. In all instances, the B3LYP functional in combination with the 6-311+g(d,p) basis set were employed. All MD runs were performed by using the GROMACS package [69]. The GAFF force field was adopted to describe both intramolecular and intermolecular interactions of the solutes [70]. The TIP3P force field was used to describe water molecules [71]. In order to refine the solute electrostatic interactions, RESP charges [72] were computed at the B3LYP/6-311 + g(d,p) level of theory. Electrostatic interactions were treated by using the particle–mesh Ewald (PME) method with a grid spacing of 0.16 Å and a spline interpolation of order 4 [73]. The cross-interactions for Lennard–Jones terms were calculated using the Lorentz–Berthelot mixing rules and intramolecular interactions between atom pairs separated up to three bonds were excluded.

A single molecule was dissolved in a cubic box containing at least 2500 water molecules. In MD${}_{\mathrm{VS}}$, the position of virtual sites was determined by performing a molecular orbital localization by means of the Boys procedure [74]. In particular, a pair of VS was assigned to each Oxygen atom (green spheres in Figure 2). The charge of each oxygen atom was uniformly split between its virtual sites.

The molecular systems were initially brought to 0 K applying the steepest descent minimization procedure and then heated to 298.15 K in an NVT ensemble using the velocity-rescaling [75] method with an integration time step of 0.1 fs and a coupling constant of 0.1 ps for 200 ps. A 500 ps long NPT run was performed, using the Parrinello–Rahman barostat and a coupling constant of 1.0 ps, to obtain a uniform distribution of molecules in the box and for thermalization purposes. Finally, 30 ns long MD production runs were carried out in the NVT ensemble, with a 0.1 fs time step.

A snapshot every 100 ps was extracted in order to obtain a total of 300 uncorrelated snapshots for each system. From each snapshot, a solute-centered sphere with radius 15 Å was cut. For each obtained droplet, the solute excitation energies were computed using the polarizable QM/FQ model [35,36,42]. The QM portion was treated at the B3LYP/6-311+g(d,p) level. The FQ SPC parametrization proposed by Rick et al [59]. was exploited. All computed absorption spectra were convoluted with a Gaussian band shape with a Full Width at Half Maximum (FWHM) of 0.37 eV, and then averaged to obtain the final spectrum.

### 2.2. MD Analysis

The analysis of each MD trajectory was performed by using the TRAVIS package [76]. Two different observables are presented and discussed: the radial distribution function (RDF) and the dihedral distribution function (DDF). Then, hydration patters defining HB interactions are discussed for the atomic sites highlighted in Figure 2. All results are commented for both MD${}_{\mathrm{noVS}}$ and MD${}_{\mathrm{VS}}$.

#### 2.2.1. Conformational Analysis Based on MD Simulations

**Figure 3:**
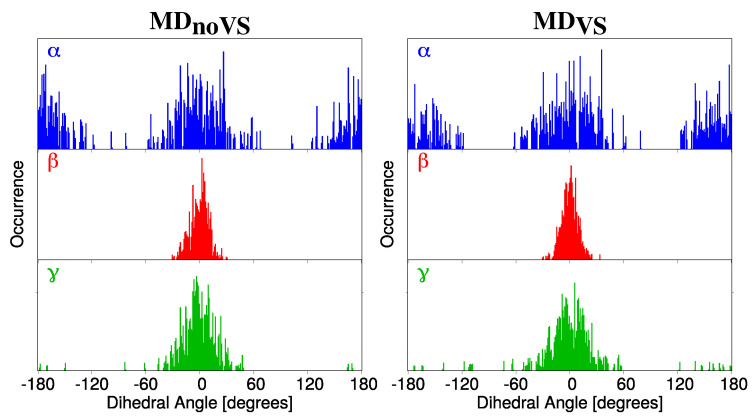
DDF of α (top,blue), β (middle,red) and γ (bottom,green) dihedral angles of Myricetin (**M**) as obtained from MD${}_{\mathrm{noVS}}$ (**left**) and MD${}_{\mathrm{VS}}$ (**right**).

DDFs were extracted for four dihedral angles (α, β, γ, δ, see Figure 2 for their definition) from both MD${}_{\mathrm{VS}}$ and MD${}_{\mathrm{noVS}}$. α, β, and γ represent the dihedral angle between the molecular plane and the selected hydroxyl groups, whereas δ describes the relative orientation of the phenyl group with respect to the main molecular plane (see Figure 2). The α, β, γ DDFs for Myricetin **M** as obtained from both MD${}_{\mathrm{noVS}}$ and MD${}_{\mathrm{VS}}$ are reported in Figure 3. The same DDFs for the other flavonoids are reported in Figures S1–S3 in the Supplementary Materials (SM).

The α DDF depicted in Figure 3 is centered at 0 and 180 degrees, with a range of variability of about 50 degrees. This indicates that the hydroxyl group involved in the definition of α presents a high rotation freedom. In addition, all studied molecules present similar α DDFs (see Figures S1–S3 in the SM), showing that the different number of OH groups on the phenyl moiety does not affect α conformational stability. The β and γ DDFs are characterized by a sharp distribution centered at zero degrees. Such a low rotational freedom of the selected hydroxyl groups is due to the intramolecular interaction which is established with the carboxyl Oxygen atom (O3, see Figure 2). In addition, the narrower spreading of β as compared to γ can be attributed to the highest stability of the six-term ring with respect to the five-term ring formed for the two angles, respectively. Similarly to α, the distributions of both β and γ angles are not affected by the different number of OH groups in the phenyl moiety (see Figures S1–S3 in the SM). As a final comment on α, β, and γ, we note that a qualitatively analog conformational analysis can be obtained by using both MD${}_{\mathrm{noVS}}$ and MD${}_{\mathrm{VS}}$ (left and right panels of Figure 3).

**Figure 4:**
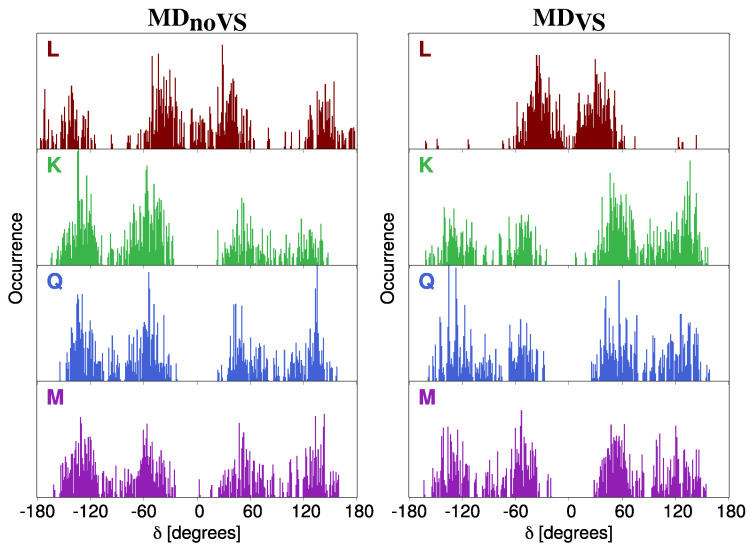
DDF of δ dihedral angle of **L** (brown), **K** (green), **Q** (blue) and **M** (purple) as obtained from MD${}_{\mathrm{noVS}}$ (**left**) and MD${}_{\mathrm{VS}}$ (**right**).

In Figure 4, δ DDFs for all studied molecules are graphically depicted. The distribution of δ dihedral angle gives insight on the planarity of the molecules because it represents the relative orientation of the two main portions of the system. We first note that δ DDFs for **K**, **Q**, and **M** are not characterized by a main preferential angle, and two different configurations can be identified, centered at ±60 and ±120 degrees. Therefore, for such systems, the two main portions never lie on the same plane. Such a consideration is valid for both MD${}_{\mathrm{noVS}}$ and MD${}_{\mathrm{VS}}$. A different picture arises for **L**δ DDF. In particular, in case of MD${}_{\mathrm{noVS}}$, the same preferential angles reported for the other molecules can be identified; however, the planar configuration is also sampled. When a more accurate description of the directionality of molecule–water interactions is taken into account by including virtual sites in the MD (MD${}_{\mathrm{VS}}$), the δ DDF has two main peaks centered at about ±60, thus showing largest molecular stiffness. Therefore, the absence of the OH group involved in the definition of γ dihedral angle significantly affects the rigidity of the molecule.

#### 2.2.2. Hydration Pattern

**Figure 5:**
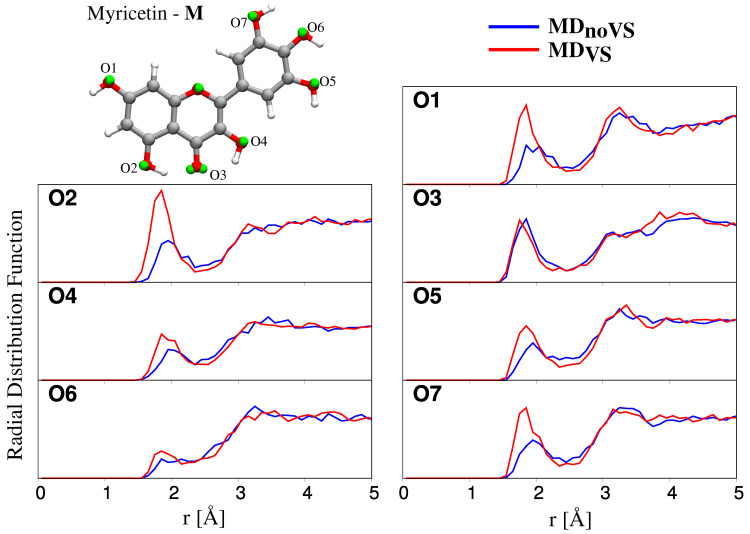
Radial distribution functions (RDFs) between Oxygen atoms of Myricetin and water Hydrogen atoms, as obtained from MD${}_{\mathrm{noVS}}$ (blue) and MD${}_{\mathrm{VS}}$ (red) runs.

All studied flavonoids are characterized by several hydroxyl groups, which can strongly interact with the surrounding water molecules via HBs. In order to study hydration patterns in aqueous solutions, both MD${}_{\mathrm{noVS}}$ and MD${}_{\mathrm{VS}}$ were analyzed by extracting RDFs between flavonoids’ Oxygen atoms and water Hydrogen atoms (Hw). Myricetin RDFs for all interacting sites are depicted in Figure 5. We focus on this system because it is characterized by the largest number of hydroxyl groups; similar data for Luteolin, Kaempferol, and Quercetin are collected in Figures S4–S6 in the SM.

All RDFs reported in Figure 5 show a peak at about 1.8 Å, of which the intensity varies for the different oxygen atoms. In particular, in most cases, a strong HB interaction is present (high intensity peak), whereas, for O6, a weaker interaction is reported in both MD${}_{\mathrm{noVS}}$ and MD${}_{\mathrm{VS}}$. Such a behavior can be explained by considering that the O6 hydroxyl group is involved in HB interactions with both O5 and O7 hydroxyl groups. This is confirmed by the fact that O6 RDFs computed for the other flavonoids do not show any significantly lower intensity with respect to RDFs related to the other Oxygen atoms (see Figures S4–S6 in SM).

The inclusion of off-site VSs has two main effects: the intensities of the RDF first peaks increase and their maxima are located at shorter *r* distances, thus indicating that a stronger solute–solvent HB interaction is described. The only notable exception is O3; however, such a behavior is not unexpected because O3 is involved in intramolecular HB between O2 and O4 hydroxyl groups. The inclusion of VSs better describes such an interaction, without affecting solute–solvent HBs.

To further analyze hydration patterns, we also computed the number of water molecules interacting with each flavonoid molecule, by extracting from MD runs the running coordinating number (RCN), see Table 1. The latter is the integral of the RDF first peak, and is related to the average number of water molecules in the first solvation shell interacting with the selected oxygen site. We first notice that, for all molecules, each oxygen site is bonded to water by at least one HB interaction; the only notable exception is O6 of myricetin, thus further confirming the comments above. Overall, the number of HBs is larger for MD${}_{\mathrm{VS}}$ than MD${}_{\mathrm{noVS}}$. This is again not surprising, and it is due to the fact that the inclusion of VSs allows for a refined, and more physically consistent, description of flavonoid–water HB interactions. In addition, a different picture arises for O3; in this case, a decrease in the number of hydrogen-bonded water molecules is reported for all the studied molecules. In fact, the inclusion of VSs leads to a stronger intramolecular HB interaction, with a consequent decrease of intermolecular HBs with water molecules.

### 2.3. Excitation Energies

**Figure 6:**
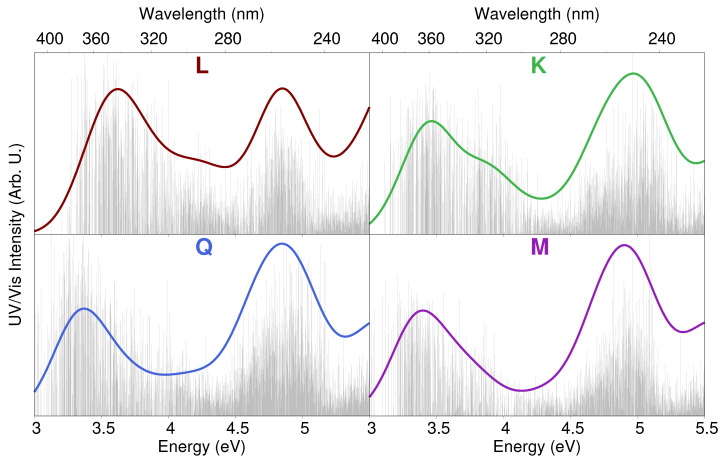
QM/FQ UV/Vis stick spectra of each studied flavonoid computed on 300 snapshots extracted from MD${}_{\mathrm{noVS}}$. Convoluted QM/FQ spectra are also plotted in color.

QM/FQ excitation energies were calculated on 300 uncorrelated snapshots extracted from MD trajectories; such a number was chosen to assure the convergence of the final spectra. All calculations were performed at the TD-B3LYP/6-311 + G(d,p) level, and the first 20 excited states were computed. QM/FQ raw data recovered from each set of snapshots are reported as stick spectra in Figure 6 for the case of MD${}_{\mathrm{noVS}}$runs. As it can be noticed, a large variability both in band intensities and energies is predicted for all flavonoids, similarly to what has already been reported for other systems dissolved in aqueous solution [77,78,79]. Similar stick spectra were obtained from snapshots extracted from MD${}_{\mathrm{VS}}$ (see Figure S7 in the SM). Stick data were then convoluted by using a Gaussian function (with FWHM = 0.37 eV), and averaged to obtained the final computed UV/Vis spectra, which are also depicted in Figure 6.

**Figure 7:**
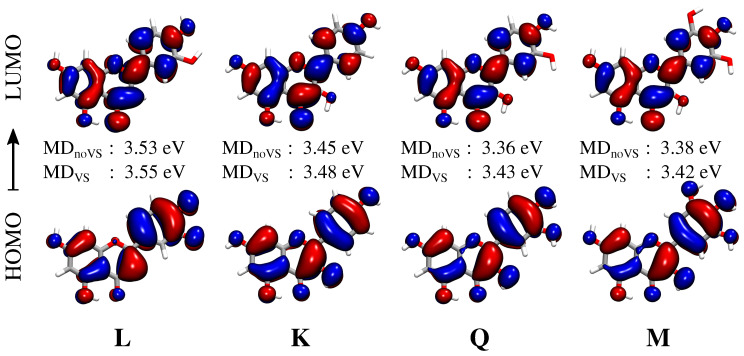
Molecular Orbitals (MOs) involved in the first electronic transition of each flavonoid. The corresponding excitation energies are also given for both MD${}_{\mathrm{VS}}$ and MD${}_{\mathrm{noVS}}$. Isovalue: 0.02.

The convoluted spectrum of each flavonoid is characterized by two main bands. The first is placed at about 3.4 eV (364 nm) for **K**, **Q** and **M**, whereas for **L** it is blueshifted by about 0.15 eV (349 nm); the second band is centered in the region 4.7–5 eV (264–248 nm) for all systems. For **K**, **Q**, and **M**, the spectrum is dominated by the second peak, whose intensity is almost twice that of the first band. A different situation is reported for **L**, for which the two bands have almost equal intensities. The first band at about 3.4–3.6 eV (364–344 nm) is due to a pure HOMO–LUMO transition, whereas the second band results from a combination of several excitations. In order to investigate the nature of the first electronic transition, the involved molecular orbitals (MOs) are plotted in Figure 7. We see that the first excitation can easily be identified as a $\pi -\pi^{*}$ transition. In particular, we note that the carbonyl Oxygen atom (O4) takes part into the electronic excitation. Therefore, the differences between **L** and the other flavonoids reported for the first transition can be ascribed to the fact that in **K**, **Q**, and **M**, O3 is hydrogen bonded to the O4 hydroxyl group (see Figure 7, right), whereas in **L** O3 is free to form a (weak) HB interaction with the surrounding water molecules. As a consequence, the $\pi -\pi^{*}$ excitation is redshifted by about 0.15 eV for **K**, **Q**, and **M**. Figure 7 also reports excitation energies of the first electronic transition for both MD${}_{\mathrm{noVS}}$ and MD${}_{\mathrm{VS}}$. Moving from **K** to **Q** and **M**, a redshift is described by both MDs. This behavior can be explained by considering that the number of hydroxyl groups increases moving from **K** to **M**. As a consequence, a larger number of HB sites with the surrounding water molecules are involved, yielding a redshift which is typically observed for $\pi -\pi^{*}$ transitions [38,80,81,82,83]. Such a redshift is not present in **Q** and **M** because the bridge OH group in M is only weakly involved in HB interactions with the solvent (see Table 1). We also note that MD${}_{\mathrm{VS}}$ always predicts higher excitation energies than MD${}_{\mathrm{noVS}}$. This is not surprising and it is once again related to the reduced number of water molecules hydrogen bonded to O3 (see Table 1).

**Figure 8:**
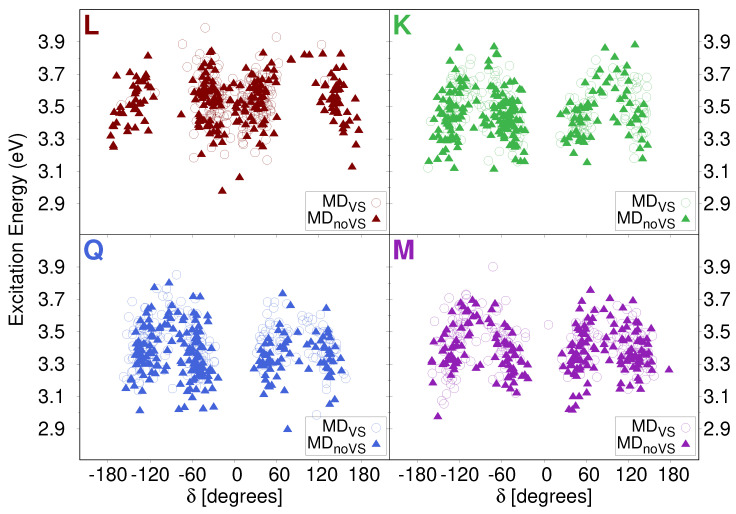
QM/FQ Excitation Energies of the first electronic transition of each studied flavonoid as a function of δ dihedral angle.

As mentioned above, a large spreading in both energies and intensities is reported in stick spectra depicted in Figure 6. In order to investigate the origin of such a variability, we studied how the excitation energy depends on the dihedral angles previously analyzed (see Section 2.2.1). In particular, Figure 8 reports computed excitation energies of the first transition as a function of the δ dihedral angle (see Figure 2 for its definition). Similar plots for α, β, and γ are given in Figures S8–S10 in the SM. We see that the computed values are clusterized around the most populated dihedral angles for both MD runs (see Figure 4). In addition, independently from the value of the dihedral angle, computed energies span the region between 2.9 and 3.9 eV. This demonstrates that the origin of the large variability reported in the stick spectra is the spatial arrangement of water molecules around the solutes.

**Figure 9:**
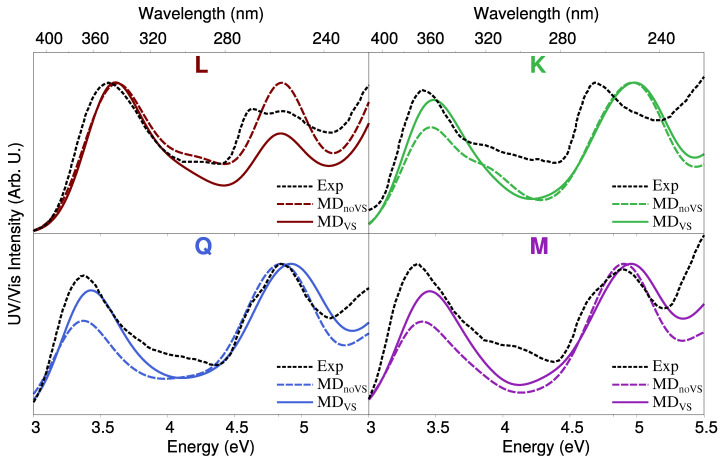
Computed absorption spectra of each studied flavonoid in aqueous solution obtained by using QM/FQ coupled with both MD${}_{\mathrm{VS}}$ and MD${}_{\mathrm{noVS}}$. Experimental UV/Vis spectra reproduced from Refs. [28,67] are also reported.

We finally move to the comparison between computed and experimental spectra, the latter reproduced from Refs. [28,67]. Such a comparison is shown in Figure 9. The experimental absorption spectrum of each molecule is characterized by two main peaks, the first lying at about 3.4 eV (365 nm) for **K**, **Q**, and **M** and 3.6 eV (345 nm) for **L**. The second peak is placed in the region 4.7–5 eV, and has a different shape for each flavonoid. In particular, for **L** and **M**, two peaks are present, probably due to vibronic coupling [84,85,86]. In addition, for **L** and **Q**, the first peak is the most intense one, whereas the opposite holds for **K**. For **M**, the intensities of the two bands almost coincide.

The agreement between QM/FQ and experimental spectra is excellent for all flavonoids, especially if QM/FQ is combined with MD${}_{\mathrm{VS}}$runs. In fact, the main difference between MD${}_{\mathrm{VS}}$ and MD${}_{\mathrm{noVS}}$ results is the relative intensity of the two band. For **K**, **Q**, and **M**, MD${}_{\mathrm{noVS}}$ underestimates the intensity of the first transition with respect to the experimental findings. Therefore, a refined modeling of intermolecular interactions by means of off-site VSs seems to be crucial to achieve an accurate reproduction of the general shape of the experimental spectra. The only notable exception is **L**, for which the experimental second band lies in between MD${}_{\mathrm{noVS}}$ and MD${}_{\mathrm{VS}}$. However, the overall agreement between the experimental and computed spectra is not compromised.

## 3. Summary and Conclusions

In this work, UV-Vis absorption spectra of four flavonoids, namely luteolin, kaempferol, quercetin, and myricetin, dissolved in aqueous solution have been simulated by exploiting a fully polarizable QM/FQ approach combined with MD simulations, performed by either including or discarding off-site VSs. For all investigated molecules, MD${}_{\mathrm{noVS}}$ and MD${}_{\mathrm{VS}}$ sample the configurational space in a similar way, whereas a different description of solute–solvent HB interactions arises. This behavior is not unexpected and it is due to a refined description of HB interactions obtained by including VSs in MD runs.

The QM/FQ spectrum of each flavonoid has been computed on a set of uncorrelated snapshots extracted from both MD trajectories. The observed differences between the various systems have been discussed in light of solute–solvent HB interactions arising from MD runs, showing that they significantly affect computed spectra. In particular, the presence of a larger number of potential HB sites, as in case of **Q** and **M** as compared to **L** and **K**, produces a redshift of the first electronic excitation, which has a $\pi -\pi^{*}$ nature. Finally, the comparison between computed and experimental spectra shows that MD${}_{\mathrm{VS}}$ provides the best agreement, probably due to better configurational sampling and HB description.

The agreement with experiments, in particular for luteolin, might be increased by exploiting polarizable force fields (instead of a standard fixed-charges one) in MD simulations, i.e., by performing a so-called polarizable MD simulation [87,88]. In this way, a more physically consistent way of describing the evolution of solute–solvent interactions could be obtained. In addition, off-site charges introduced in MD${}_{\mathrm{VS}}$ runs might be included in polarizable QM/MM calculations by resorting to polarizable force fields defined in terms of both fluctuating charges and fluctuating dipoles, similarly to what has recently been developed by some of the present authors [58,89,90]. Finally, in this work, the QM–MM coupling has been limited to the account of electrostatic solute–water interactions. The inclusion of non-electrostatic terms, i.e., Pauli repulsion and dispersion, might improve the agreement between computed and experimental data, in light of similar studies recently reported by some of us [91,92]. 

## Figures and Tables

**Table 1:** Running coordination number (RCN) of the studied Oxygen sites of the different flavonoids as obtained from MD${}_{\mathrm{noVS}}$ and MD${}_{\mathrm{VS}}$ (in parentheses). See Figure 2 for atom labeling.

Site	L	K	Q	M
O1	1.1 (1.5)	1.4 (1.6)	1.0 (1.6)	1.2 (1.5)
O2	1.3 (1.9)	1.6 (1.7)	1.2 (1.8)	1.0 (1.6)
O3	1.8 (1.6)	1.4 (1.1)	1.4 (1.2)	1.3 (1.2)
O4	– (–)	1.1 (1.3)	1.1 (1.4)	0.8 (1.1)
O5	1.0 (1.4)	– (–)	– (–)	0.9 (1.2)
O6	0.9 (1.3)	1.3 (1.7)	0.9 (1.0)	0.2 (0.5)
O7	– (–)	– (–)	1.0 (1.4)	1.1 (1.3)

## Supplementary Materials

The following are available online. Figures S1–S3: α, β and γ DDFs for L, K, and M; Figures S4–S6: RDFs between flavonoids’ Oxygen atoms and water Hydrogen atoms for L, K, and M; Figure S7: UV/Vis QM/FQ stick spectra computed on the various snapshots extracted from MD${}_{\mathrm{VS}}$; Figures S8–S10: excitation energies of the first excitation for each flavonoid as a function of α, β, and γ dihedral angles.

## References

[1] Harborne J.B., Marby H., Marby T. (1988). The Flavonoids.

[2] Kapešová J., Petrásková L., Markošová K., Rebroš M., Kotik M., Bojarová P., Křen V. (2019). Bioproduction of quercetin and rutinose catalyzed by rutinosidase: Novel concept of “solid state biocatalysis”. Int. J. Mol. Sci..

[3] Burak M., Imen Y. (1999). Flavonoids and their antioxidant properties. Turkiye Klin. Tip Bil. Derg..

[4] Casta eda Ovando A., de Lourdes Pacheco-Hernández M., Pez-Hern ndez M.E., Gal n Vidal C.A.S. (2009). Chemical studies of anthocyanins: A review. Food Chem..

[5] Lee Y.K., Yuk D.Y., Lee J.W., Lee S.Y., Ha T.Y., Oh K.W., Yun Y.P., Hong J.T. (2009). (-)-Epigallocatechin-3-gallate prevents lipopolysaccharide-induced elevation of beta-amyloid generation and memory deficiency. Brain Res..

[6] Panche A., Diwan A., Chandra S. (2016). Flavonoids: An overview. J. Nutr. Sci..

[7] Metodiewa D., Kochman A., Karolczak S. (1997). Evidence for antiradical and antioxidant properties of four biologically active N, N-Diethylaminoethyl ethers of flavaone oximes: A comparison with natural polyphenolic flavonoid rutin action. IUBMB Life.

[8] Hayashi T., Sawa K., Kawasaki M., Arisawa M., Shimizu M., Morita N. (1988). Inhibition of cow’s milk xanthine oxidase by flavonoids. J. Nat. Prod..

[9] Ngwa W., Kumar R., Thompson D., Lyerly W., Moore R., Reid T.E., Lowe H., Toyang N. (2020). Potential of Flavonoid-Inspired Phytomedicines against COVID-19. Molecules.

[10] Das P., Majumder R., Mandal M., Basak P. (2020). In-Silico approach for identification of effective and stable inhibitors for COVID-19 main protease (Mpro) from flavonoid based phytochemical constituents of Calendula officinalis. J. Biomol. Struct. Dyn..

[11] Solnier J., Fladerer J.P. (2020). Flavonoids: A complementary approach to conventional therapy of COVID-19?. Phytochem. Rev..

[12] Robak J., Gryglewski R. (1996). Bioactivity of flavonoids. Pol. J. Pharmacol..

[13] Rice-Evans C., Miller N. (1996). Antioxidant activities of flavonoids as bioactive components of food. Biochem. Soc. Trans..

[14] Spencer J.P., Abd El Mohsen M.M., Rice-Evans C. (2004). Cellular uptake and metabolism of flavonoids and their metabolites: Implications for their bioactivity. Arch. Biochem. Biophys..

[15] Pinent M., Castell A., Baiges I., Montagut G., Arola L., Ardévol A. (2008). Bioactivity of flavonoids on insulin-secreting cells. Compr. Rev. Food Sci..

[16] Erlund I. (2004). Review of the flavonoids quercetin, hesperetin, and naringenin. Dietary sources, bioactivities, bioavailability, and epidemiology. Nutr. Res..

[17] Rohan T., Connell M. (1964). The precursors of chocolate aroma: A study of the flavonoids and phenolic acids. J. Food Sci..

[18] Samanta A., Das G., Das S.K. (2011). Roles of flavonoids in plants. Carbon.

[19] Sadighara P., Gharibi S., Jafari A.M., Khaniki G.J., Salari S. (2012). The antioxidant and Flavonoids contents of Althaea officinalis L. flowers based on their color. Avicenna J. Phytomed..

[20] Yoshida K., Oyama K., Kondo T. (2012). Chemistry of flavonoids in color development. Rec. Adv. Polyphen. Res..

[21] Kennedy J.A., Matthews M.A., Waterhouse A.L. (2002). Effect of maturity and vine water status on grape skin and wine flavonoids. Am. J. Enol. Viticult..

[22] Waterhouse A.L. (2002). Wine phenolics. Ann. N. Y. Acad. Sci..

[23] Lairon D., Amiot M.J. (1999). Flavonoids in food and natural antioxidants in wine. Curr. Opin. Lipidol..

[24] Fernandes I., Pérez-Gregorio R., Soares S., Mateus N., De Freitas V. (2017). Wine flavonoids in health and disease prevention. Molecules.

[25] Miyagi Y., Miwa K., Inoue H. (1997). Inhibition of human low-density lipoprotein oxidation by flavonoids in red wine and grape juice. Am. J. Cardiol..

[26] Tapas A.R., Sakarkar D., Kakde R. (2008). Flavonoids as nutraceuticals: A review. Trop. J. Pharm. Res..

[27] Takahashi A., Ohnishi T. (2004). The significance of the study about the biological effects of solar ultraviolet radiation using the Exposed Facility on the International Space Station. Biol. Sci. Space.

[28] Biler M., Biedermann D., Valentová K., Křen V., Kubala M. (2017). Quercetin and its analogues: Optical and acido–basic properties. Phys. Chem. Chem. Phys..

[29] Duan Y. (2014). Ultraviolet-visible spectrum characterizations of quercetin in aqueous ethanol solution with different pH values. J. Chem. Pharm. Res..

[30] Dall’Acqua S., Miolo G., Innocenti G., Caffieri S. (2012). The photodegradation of quercetin: Relation to oxidation. Molecules.

[31] Cerrato A., De Santis D., Moresi M. (2002). Production of luteolin extracts from Reseda luteola and assessment of their dyeing properties. J. Sci. Food Agric..

[32] M Calderon-Montano J., Burgos-Morón E., Pérez-Guerrero C., López-Lázaro M. (2011). A review on the dietary flavonoid kaempferol. Mini-Rev. Med. Chem..

[33] McDonald M.S., Hughes M., Burns J., Lean M.E., Matthews D., Crozier A. (1998). Survey of the free and conjugated myricetin and quercetin content of red wines of different geographical origins. J. Agric. Food Chem..

[34] Fang F., Li J.M., Pan Q.H., Huang W.D. (2007). Determination of red wine flavonoids by HPLC and effect of aging. Food Chem..

[35] Giovannini T., Egidi F., Cappelli C. (2020). Molecular spectroscopy of aqueous solutions: A theoretical perspective. Chem. Soc. Rev..

[36] Giovannini T., Egidi F., Cappelli C. (2020). Theory and algorithms for chiroptical properties and spectroscopies of aqueous systems. Phys. Chem. Chem. Phys..

[37] Loco D., Polack É., Caprasecca S., Lagardere L., Lipparini F., Piquemal J.P., Mennucci B. (2016). A QM/MM approach using the AMOEBA polarizable embedding: From ground state energies to electronic excitations. J. Chem. Theory Comput..

[38] Aidas K., Møgelhøj A., Nilsson E.J., Johnson M.S., Mikkelsen K.V., Christiansen O., Söderhjelm P., Kongsted J. (2008). On the performance of quantum chemical methods to predict solvatochromic effects: The case of acrolein in aqueous solution. J. Chem. Phys..

[39] Tomasi J., Persico M. (1994). Molecular interactions in solution: An overview of methods based on continuous distributions of the solvent. Chem. Rev..

[40] Tomasi J., Mennucci B., Cammi R. (2005). Quantum mechanical continuum solvation models. Chem. Rev..

[41] Tomasi J., Cammi R., Mennucci B., Cappelli C., Corni S. (2002). Molecular properties in solution described with a continuum solvation model. Phys. Chem. Chem. Phys..

[42] Cappelli C. (2016). Integrated QM/Polarizable MM/Continuum Approaches to Model Chiroptical Properties of Strongly Interacting Solute-Solvent Systems. Int. J. Quantum Chem..

[43] Mennucci B. (2012). Polarizable Continuum Model. WIREs Comput. Mol. Sci..

[44] Giovannini T., Ambrosetti M., Cappelli C. (2018). A polarizable embedding approach to second harmonic generation (SHG) of molecular systems in aqueous solutions. Theor. Chem. Acc..

[45] Egidi F., Russo R., Carnimeo I., D’Urso A., Mancini G., Cappelli C. (2015). The Electronic Circular Dichroism of Nicotine in Aqueous Solution: A Test Case for Continuum and Mixed Explicit-Continuum Solvation Approaches. J. Phys. Chem. A.

[46] Lipparini F., Egidi F., Cappelli C., Barone V. (2013). The optical rotation of methyloxirane in aqueous solution: A never ending story?. J. Chem. Theory Comput..

[47] Cappelli C., Mennucci B., Monti S. (2005). Environmental effects on the spectroscopic properties of gallic acid: A combined classical and quantum mechanical study. J. Phys. Chem. A.

[48] Warshel A., Karplus M. (1972). Calculation of ground and excited state potential surfaces of conjugated molecules. I. Formulation and parametrization. J. Am. Chem. Soc..

[49] Warshel A., Levitt M. (1976). Theoretical studies of enzymic reactions: Dielectric, electrostatic and steric stabilization of the carbonium ion in the reaction of lysozyme. J. Mol. Biol..

[50] Senn H.M., Thiel W. (2009). QM/MM methods for biomolecular systems. Angew. Chem. Int. Ed..

[51] Lin H., Truhlar D.G. (2007). QM/MM: What have we learned, where are we, and where do we go from here?. Theor. Chem. Acc..

[52] Curutchet C., Muñoz-Losa A., Monti S., Kongsted J., Scholes G.D., Mennucci B. (2009). Electronic energy transfer in condensed phase studied by a polarizable QM/MM model. J. Chem. Theory Comput..

[53] Lipparini F., Cappelli C., Scalmani G., De Mitri N., Barone V. (2012). Analytical first and second derivatives for a fully polarizable QM/classical hamiltonian. J. Chem. Theory Comput..

[54] Lipparini F., Cappelli C., Barone V. (2012). Linear response theory and electronic transition energies for a fully polarizable QM/classical hamiltonian. J. Chem. Theory Comput..

[55] Olsen J.M.H., Kongsted J. (2011). Molecular properties through polarizable embedding. Adv. Quantum Chem..

[56] Olsen J.M.H., Steinmann C., Ruud K., Kongsted J. (2015). Polarizable density embedding: A new QM/QM/MM-based computational strategy. J. Phys. Chem. A.

[57] Boulanger E., Thiel W. (2012). Solvent boundary potentials for hybrid QM/MM computations using classical drude oscillators: A fully polarizable model. J. Chem. Theory Comput..

[58] Giovannini T., Puglisi A., Ambrosetti M., Cappelli C. (2019). Polarizable QM/MM approach with fluctuating charges and fluctuating dipoles: The QM/FQFμ model. J. Chem. Theory Comput..

[59] Rick S.W., Stuart S.J., Berne B.J. (1994). Dynamical fluctuating charge force fields: Application to liquid water. J. Chem. Phys..

[60] Rick S.W., Stuart S.J., Bader J.S., Berne B. (1995). Fluctuating charge force fields for aqueous solutions. J. Mol. Liq..

[61] Rick S.W., Berne B.J. (1996). Dynamical Fluctuating Charge Force Fields: The Aqueous Solvation of Amides. J. Am. Chem. Soc..

[62] Giovannini T., Olszowka M., Cappelli C. (2016). Effective Fully Polarizable QM/MM Approach To Model Vibrational Circular Dichroism Spectra of Systems in Aqueous Solution. J. Chem. Theory Comput..

[63] Egidi F., Giovannini T., Del Frate G., Lemler P.M., Vaccaro P.H., Cappelli C. (2019). A combined experimental and theoretical study of optical rotatory dispersion for (R)-glycidyl methyl ether in aqueous solution. Phys. Chem. Chem. Phys..

[64] Giovannini T., Macchiagodena M., Ambrosetti M., Puglisi A., Lafiosca P., Lo Gerfo G., Egidi F., Cappelli C. (2019). Simulating vertical excitation energies of solvated dyes: From continuum to polarizable discrete modeling. Int. J. Quantum Chem..

[65] Macchiagodena M., Mancini G., Pagliai M., Barone V. (2016). Accurate prediction of bulk properties in hydrogen bonded liquids: Amides as case studies. Phys. Chem. Chem. Phys..

[66] Macchiagodena M., Mancini G., Pagliai M., Cardini G., Barone V. (2017). New atomistic model of pyrrole with improved liquid state properties and structure. Int. J. Quantum Chem..

[67] Naseem B., Sabri A., Hasan A., Shah S.S. (2004). Interaction of flavonoids within organized molecular assemblies of anionic surfactant. Colloids Surfaces B Biointerfaces.

[68] Frisch M.J., Trucks G.W., Schlegel H.B., Scuseria G.E., Robb M.A., Cheeseman J.R., Scalmani G., Barone V., Petersson G.A., Nakatsuji H. (2016). Gaussian 16 Revision A.03.

[69] Abrahama M.J., Murtola T., Schulz R., Pálla S., Smith J.C., Hess B., Lindahl E. (2015). GROMACS: High Performance Molecular Simulations through Multi-Level Parallelism from Laptops to Supercomputers. SoftwareX.

[70] Wang J., Wolf R.M., Caldwell J.W., Kollman P.A., Case D.A. (2004). Development and testing of a general amber force field. J. Comput. Chem..

[71] Mark P., Nilsson L. (2001). Structure and dynamics of the TIP3P, SPC, and SPC/E water models at 298 K. J. Phys. Chem. A.

[72] Bayly C.I., Cieplak P., Cornell W., Kollman P.A. (1993). A well-behaved electrostatic potential based method using charge restraints for deriving atomic charges: The RESP model. J. Phys. Chem..

[73] Darden T., York D., Pedersen L. (1993). Particle mesh Ewald: An Nlog(N) method for Ewald sums in large systems. J. Chem. Phys..

[74] Boys S. (1966). Quantum Theory of Atoms, Molecules, and the Solid State.

[75] Bussi G., Donadio D., Parrinello M. (2007). Canonical sampling through velocity rescaling. J. Chem. Phys..

[76] Brehm M., Kirchner B. (2011). TRAVIS-A Free Analyzer and Visualizer for Monte Carlo and Molecular Dynamics Trajectories. J. Chem. Inf. Model..

[77] Puglisi A., Giovannini T., Antonov L., Cappelli C. (2019). Interplay between conformational and solvent effects in UV-visible absorption spectra: Curcumin tautomers as a case study. Phys. Chem. Chem. Phys..

[78] Gómez S., Giovannini T., Cappelli C. (2020). Absorption spectra of xanthines in aqueous solution: A computational study. Phys. Chem. Chem. Phys..

[79] Di Remigio R., Giovannini T., Ambrosetti M., Cappelli C., Frediani L. (2019). Fully polarizable QM/fluctuating charge approach to two-photon absorption of aqueous solutions. J. Chem. Theory Comput..

[80] Reichardt C. (1992). Solvatochromism, thermochromism, piezochromism, halochromism, and chiro-solvatochromism of pyridinium N-phenoxide betaine dyes. Chem. Soc. Rev..

[81] Reichardt C. (1994). Solvatochromic dyes as solvent polarity indicators. Chem. Rev..

[82] Marenich A.V., Cramer C.J., Truhlar D.G., Guido C.A., Mennucci B., Scalmani G., Frisch M.J. (2011). Practical computation of electronic excitation in solution: Vertical excitation model. Chem. Sci..

[83] Duchemin I., Guido C.A., Jacquemin D., Blase X. (2018). The Bethe–Salpeter formalism with polarisable continuum embedding: Reconciling linear-response and state-specific features. Chem. Sci..

[84] Biczysko M., Bloino J., Brancato G., Cacelli I., Cappelli C., Ferretti A., Lami A., Monti S., Pedone A., Prampolini G. (2012). Integrated computational approaches for spectroscopic studies of molecular systems in the gas phase and in solution: Pyrimidine as a test case. Theor. Chem. Acc..

[85] Hodecker M., Biczysko M., Dreuw A., Barone V. (2016). Simulation of Vacuum UV Absorption and Electronic Circular Dichroism Spectra of Methyl Oxirane: The Role of Vibrational Effects. J. Chem. Theory Comput..

[86] Improta R., Santoro F., Barone V., Lami A. (2009). Vibronic model for the quantum dynamical study of the competition between bright and charge-transfer excited states in single-strand polynucleotides: The adenine dimer case. J. Phys. Chem. A.

[87] Lamoureux G., Harder E., Vorobyov I.V., Roux B., MacKerell A.D. (2006). A polarizable model of water for molecular dynamics simulations of biomolecules. Chem. Phys. Lett..

[88] Wu J.C., Piquemal J.P., Chaudret R., Reinhardt P., Ren P. (2010). Polarizable molecular dynamics simulation of Zn (II) in water using the AMOEBA force field. J. Chem. Theory Comput..

[89] Giovannini T., Riso R.R., Ambrosetti M., Puglisi A., Cappelli C. (2019). Electronic transitions for a fully polarizable qm/mm approach based on fluctuating charges and fluctuating dipoles: Linear and corrected linear response regimes. J. Chem. Phys..

[90] Giovannini T., Grazioli L., Ambrosetti M., Cappelli C. (2019). Calculation of ir spectra with a fully polarizable qm/mm approach based on fluctuating charges and fluctuating dipoles. J. Chem. Theory Comput..

[91] Giovannini T., Lafiosca P., Cappelli C. (2017). A General Route to Include Pauli Repulsion and Quantum Dispersion Effects in QM/MM Approaches. J. Chem. Theory Comput..

[92] Giovannini T., Ambrosetti M., Cappelli C. (2019). Quantum Confinement Effects on Solvatochromic Shifts of Molecular Solutes. J. Phys. Chem. Lett..

