# Supplementary material for: Simulating Absorption Spectra of Flavonoids in Aqueous Solution: A Polarizable QM/MM Study
Source: Molecules. 2020 Dec 11;25(24):5853. doi: 10.3390/molecules25245853 (PMC7764712; doi:10.3390/molecules25245853)
Supplement: Supplementary file 1 [file molecules-25-05853-s001.pdf]

# Supplementary Materials for: Simulating Absorption Spectra of Flavonoids in Aqueous Solution: a Polarizable QM/MM Study

Sulejman Skoko,[†] Matteo Ambrosetti,[†] Tommaso Giovannini,[‡] and Chiara Cappelli[*,†]

[†]*Scuola Normale Superiore, Piazza dei Cavalieri 7, 56126 Pisa, Italy.*

[‡]*Department of Chemistry, Norwegian University of Science and Technology, 7491 Trondheim, Norway*

E-mail: chiara.cappelli@sns.it

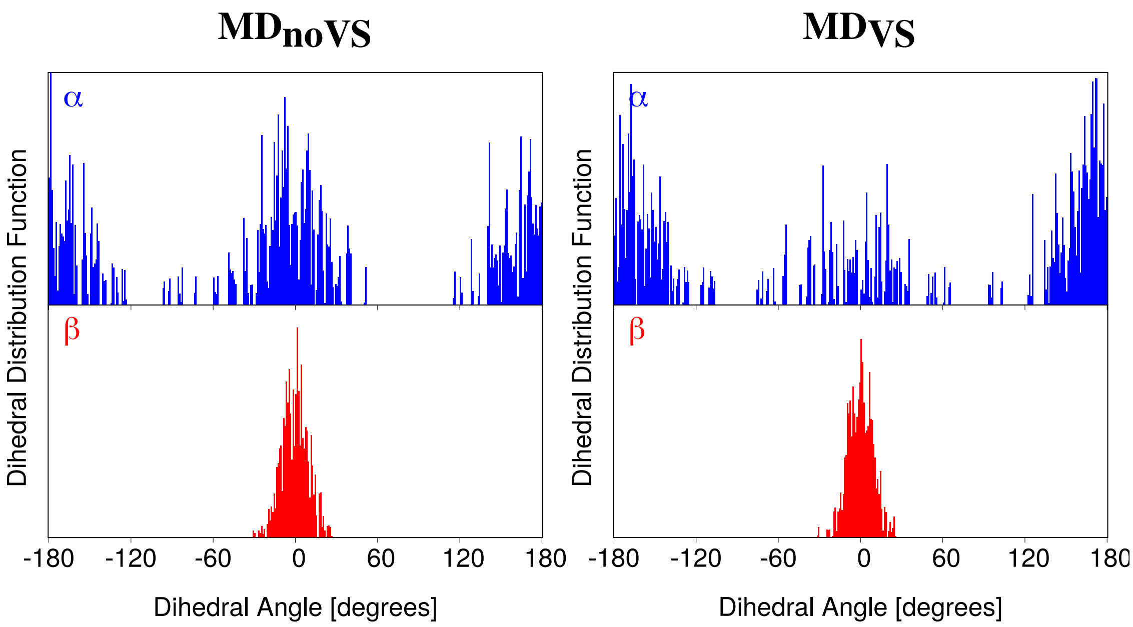


Figure S1: Dihedral distribution functions of $\alpha$ (top,blue), $\beta$ (bottom,red) dihedral angles of Luteolin (**L**) as obtained from $MD_{noVS}$ (left) and $MD_{VS}$ (right).

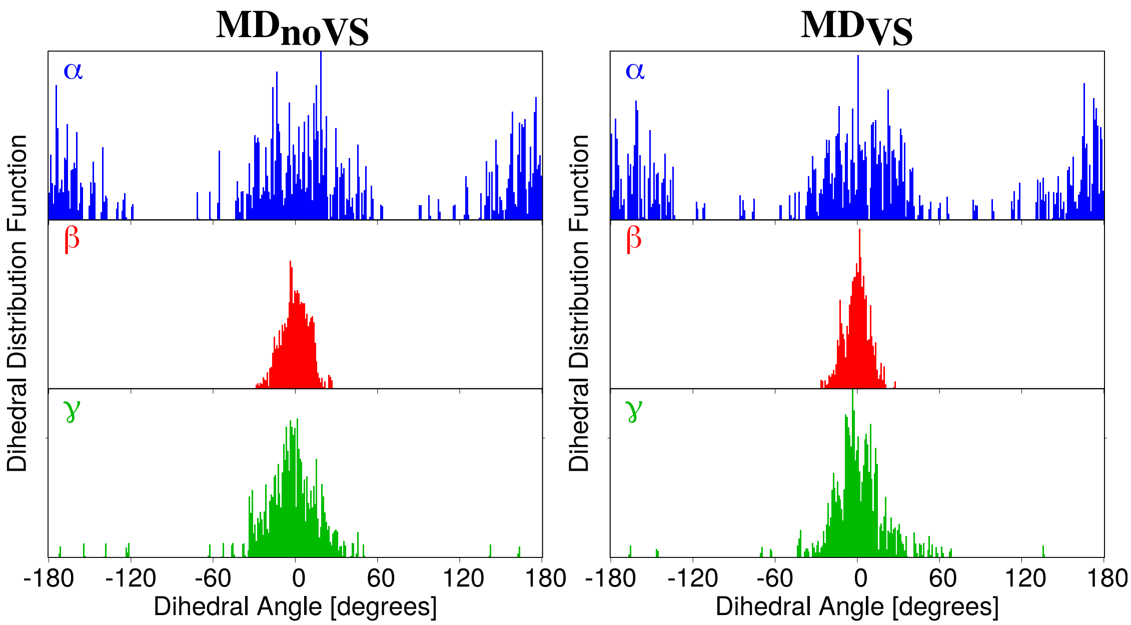


Figure S2: Dihedral distribution functions of $\alpha$ (top,blue), $\beta$ (middle,red) and $\gamma$ (bottom,green) dihedral angles of Kaempferol (**K**) as obtained from $MD_{noVS}$ (left) and $MD_{VS}$ (right).

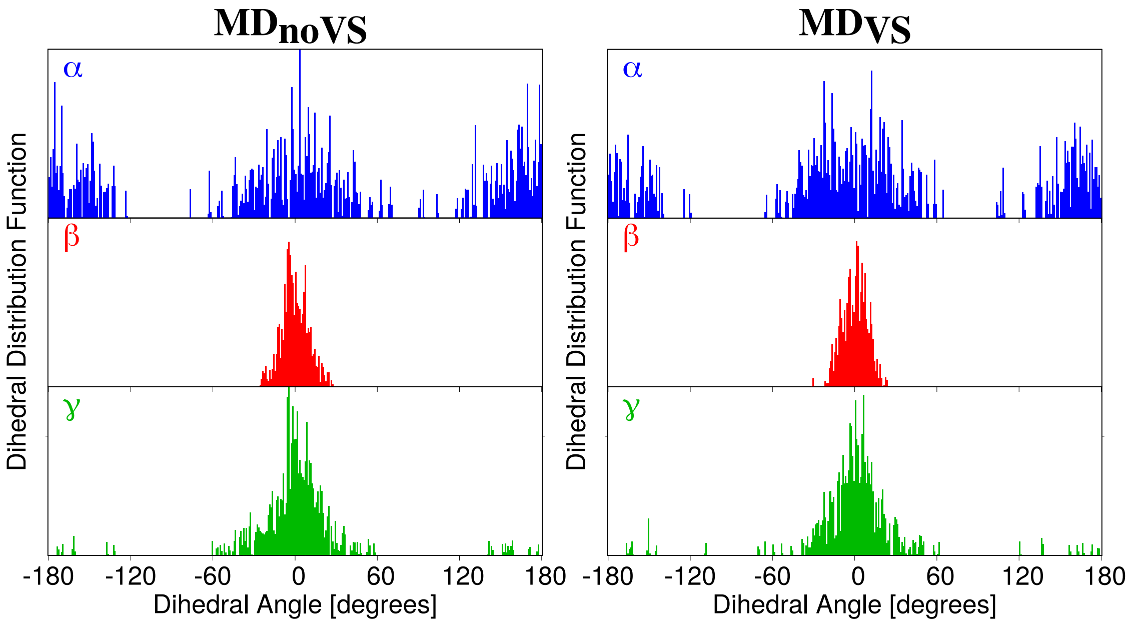


Figure S3: Dihedral distribution functions of $\alpha$ (top,blue), $\beta$ (middle,red) and $\gamma$ (bottom,green) dihedral angles of Quercetin (**Q**) as obtained from MD$_{\mathrm{noVS}}$ (left) and MD$_{\mathrm{VS}}$ (right).

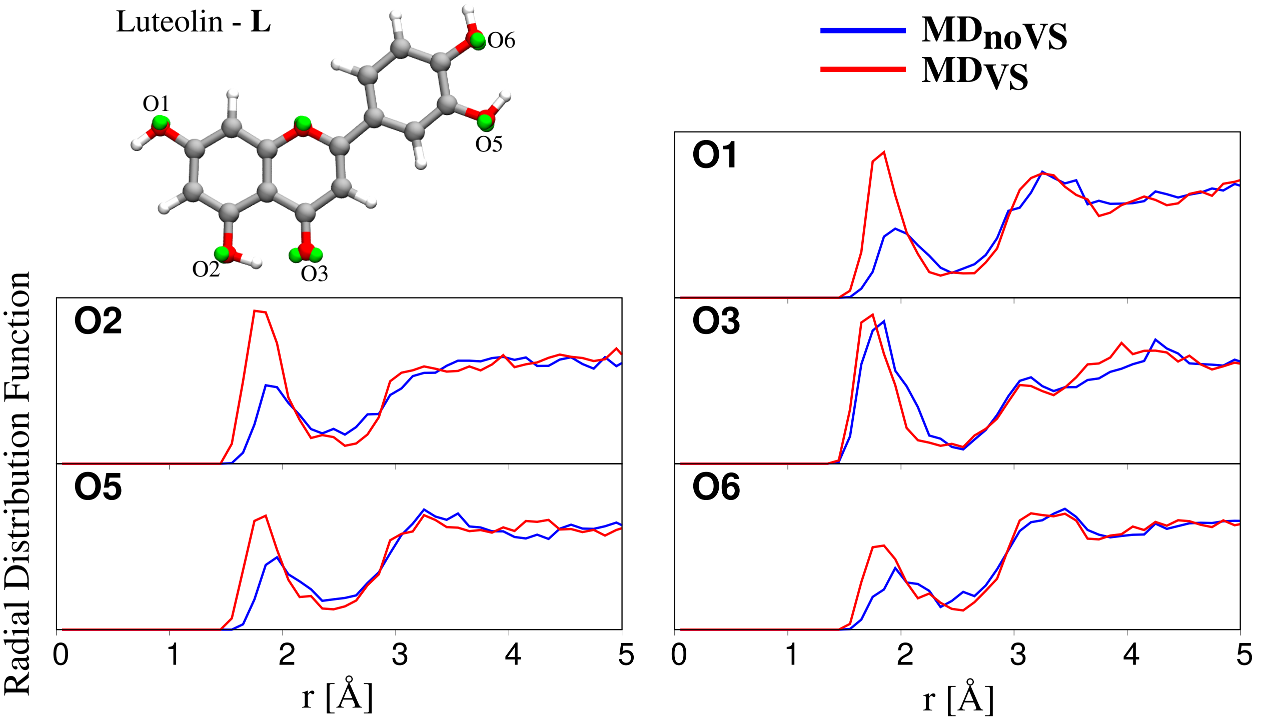


Figure S4: Radial distribution functions between selected Oxygen atoms of Luteolin and water Hydrogen atoms as obtained from MD$_{\mathrm{noVS}}$ (blue) and MD$_{\mathrm{VS}}$ (red).

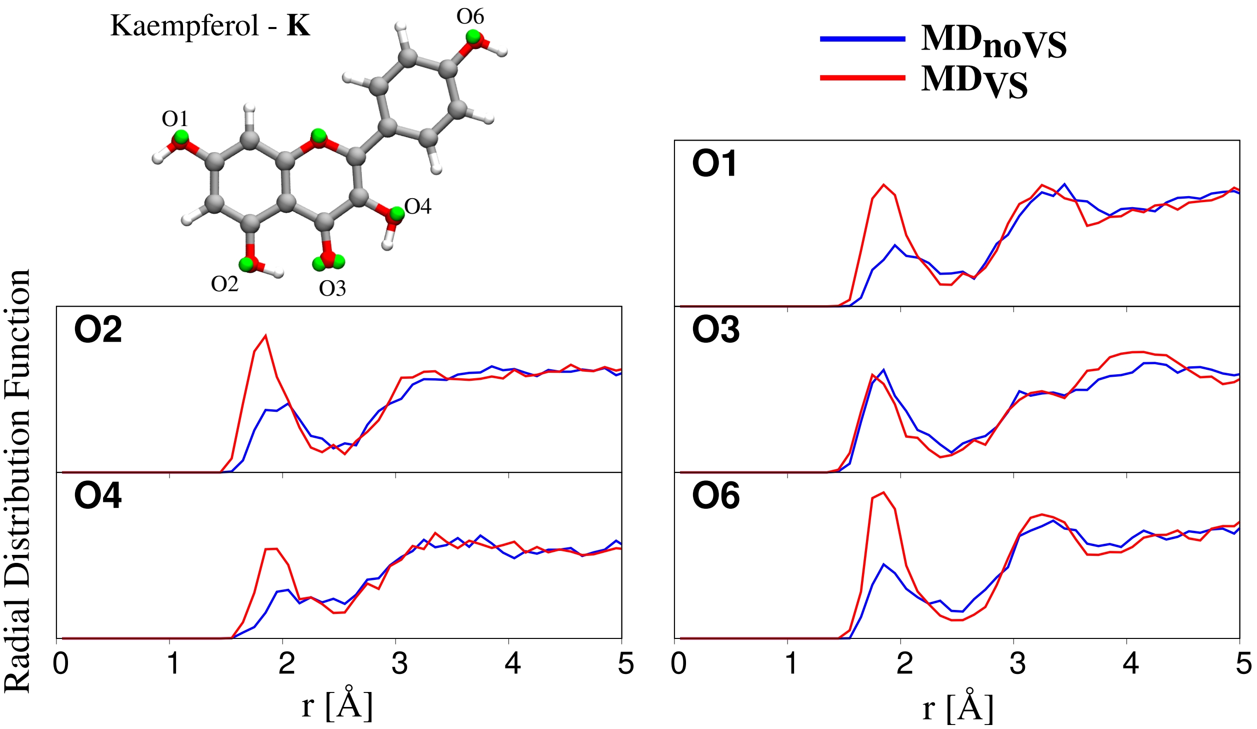


Figure S5: Radial distribution functions between selected Oxygen atoms of Kaempferol and water Hydrogen atoms as obtained from $MD_{noVS}$ (blue) and $MD_{VS}$ (red).

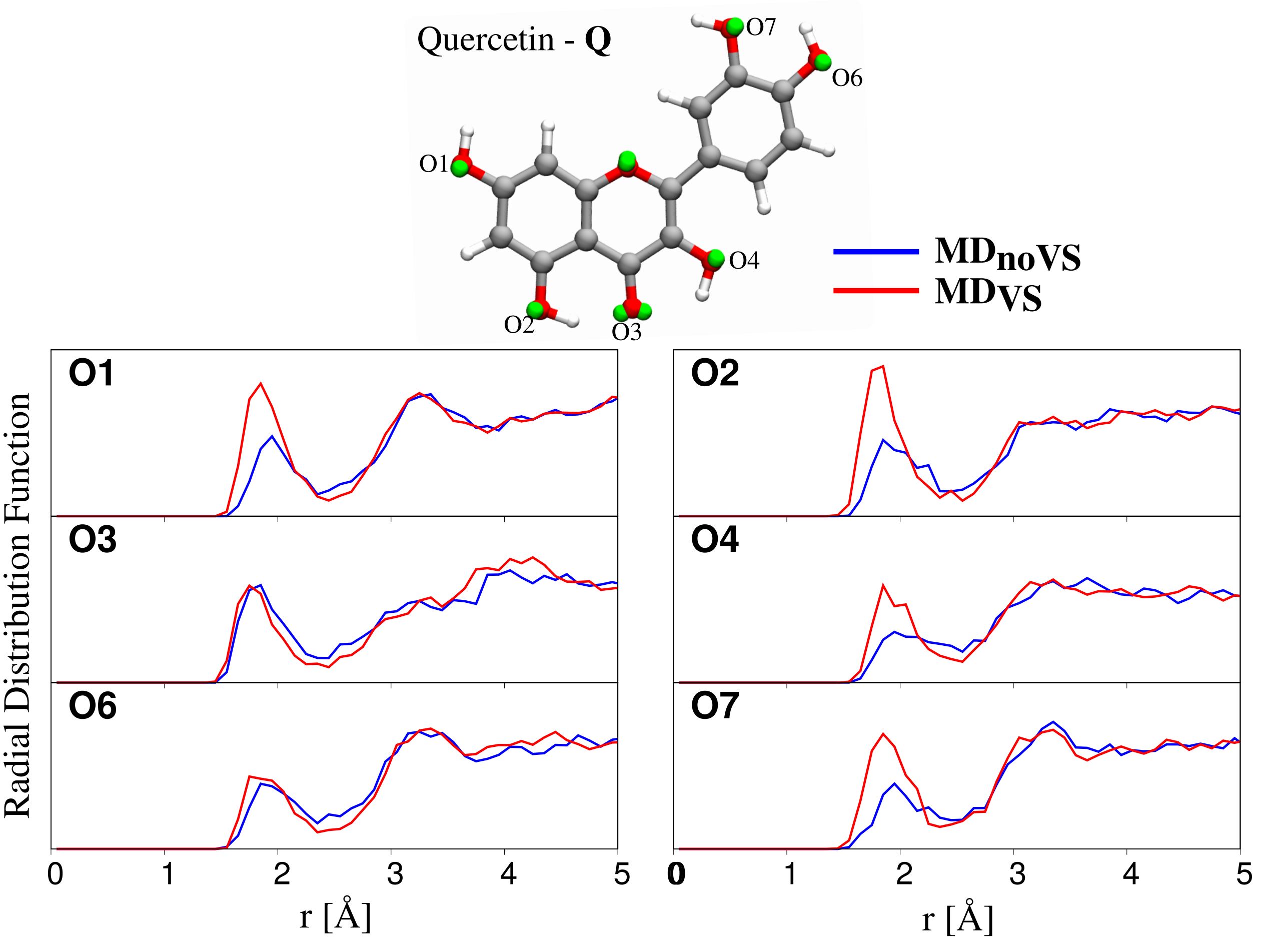


Figure S6: Radial distribution functions between selected Oxygen atoms of Quercetin and water Hydrogen atoms as obtained from $MD_{noVS}$ (blue) and $MD_{VS}$ (red).

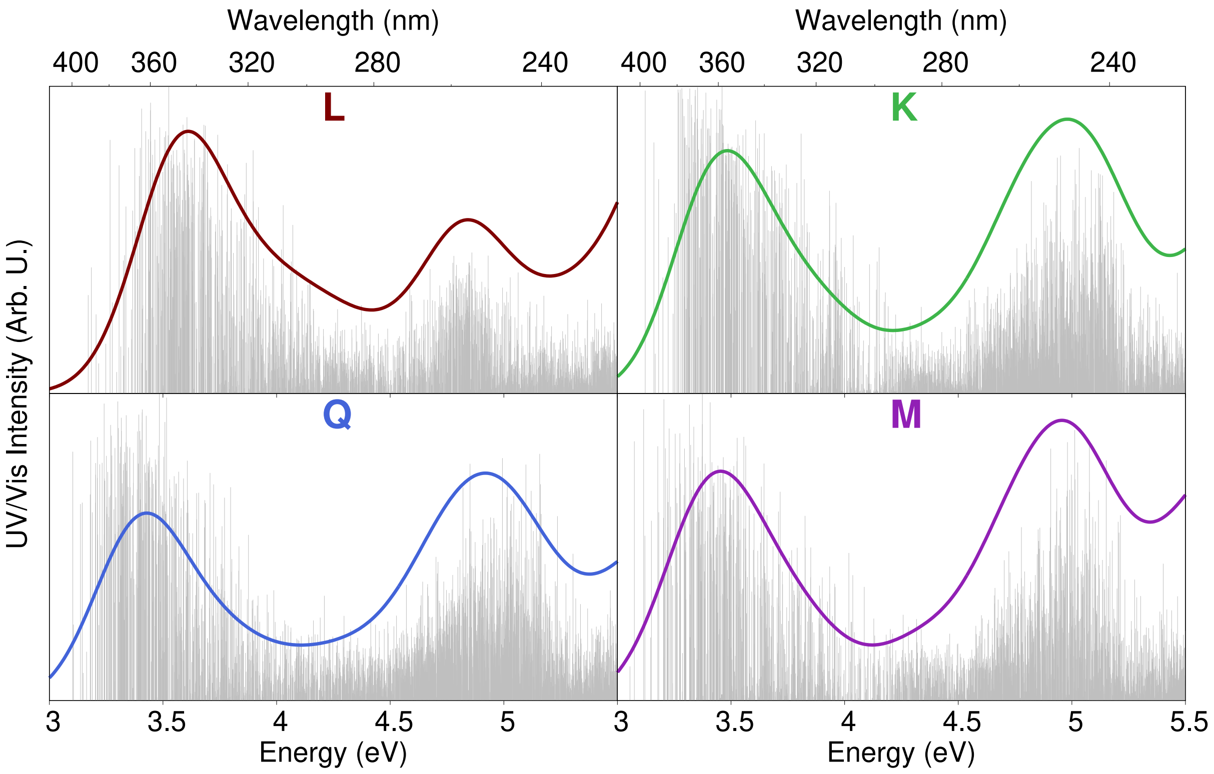


Figure S7: QM/FQ UV/Vis stick spectra computed on the snapshots extracted from $MD_{VS}$. The convoluted QM/FQ spectra are also plotted.

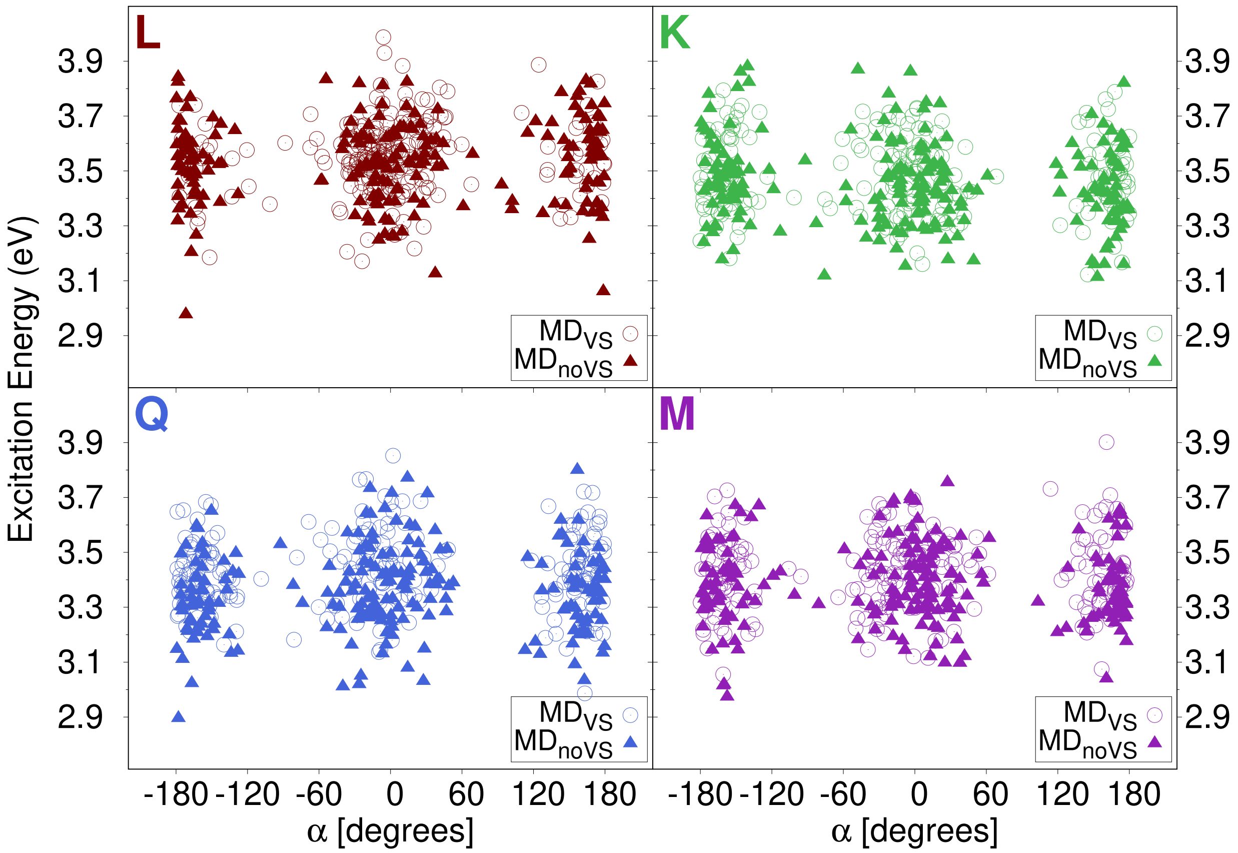


Figure S8: QM/FQ Excitation Energies of the first electronic transition as a function of $\alpha$ dihedral angle.

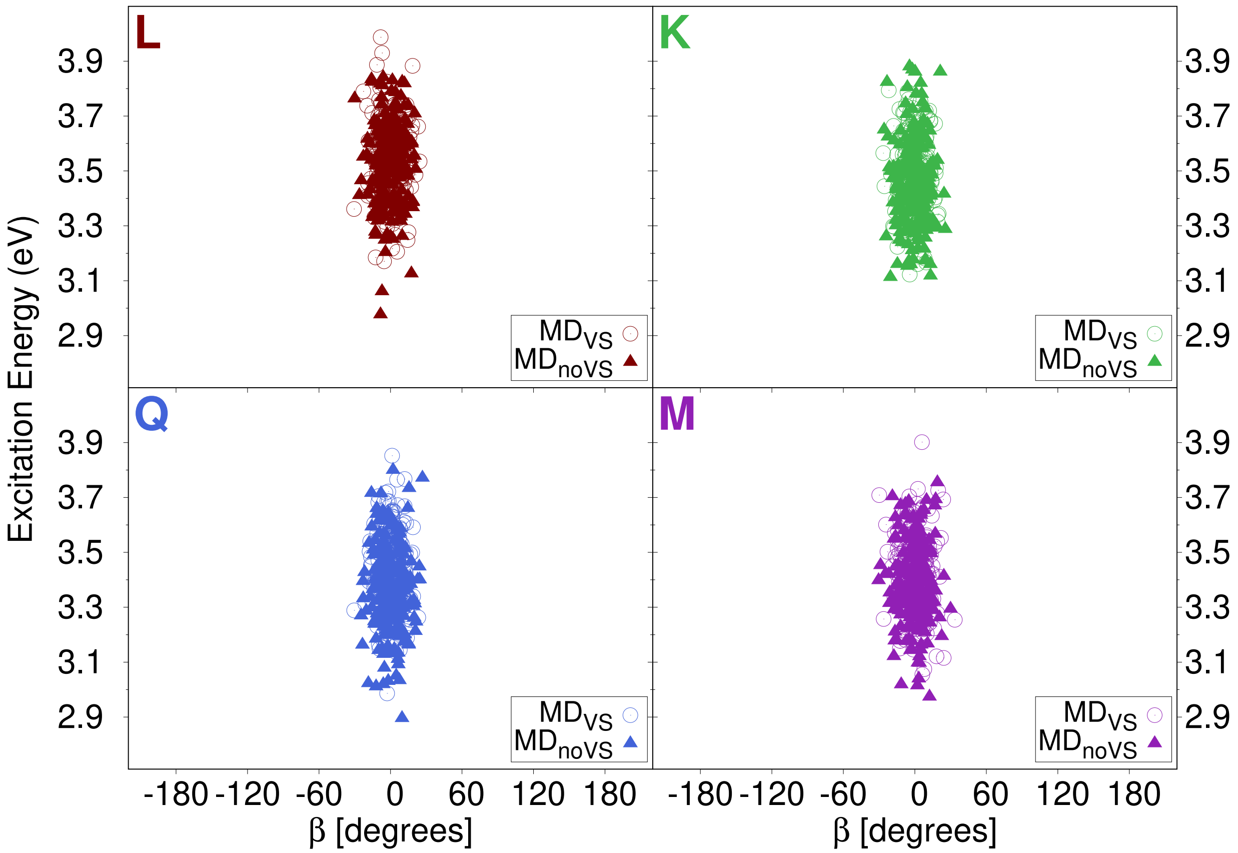


Figure S9: QM/FQ Excitation Energies of the first electronic transition as a function of $\beta$ dihedral angle.

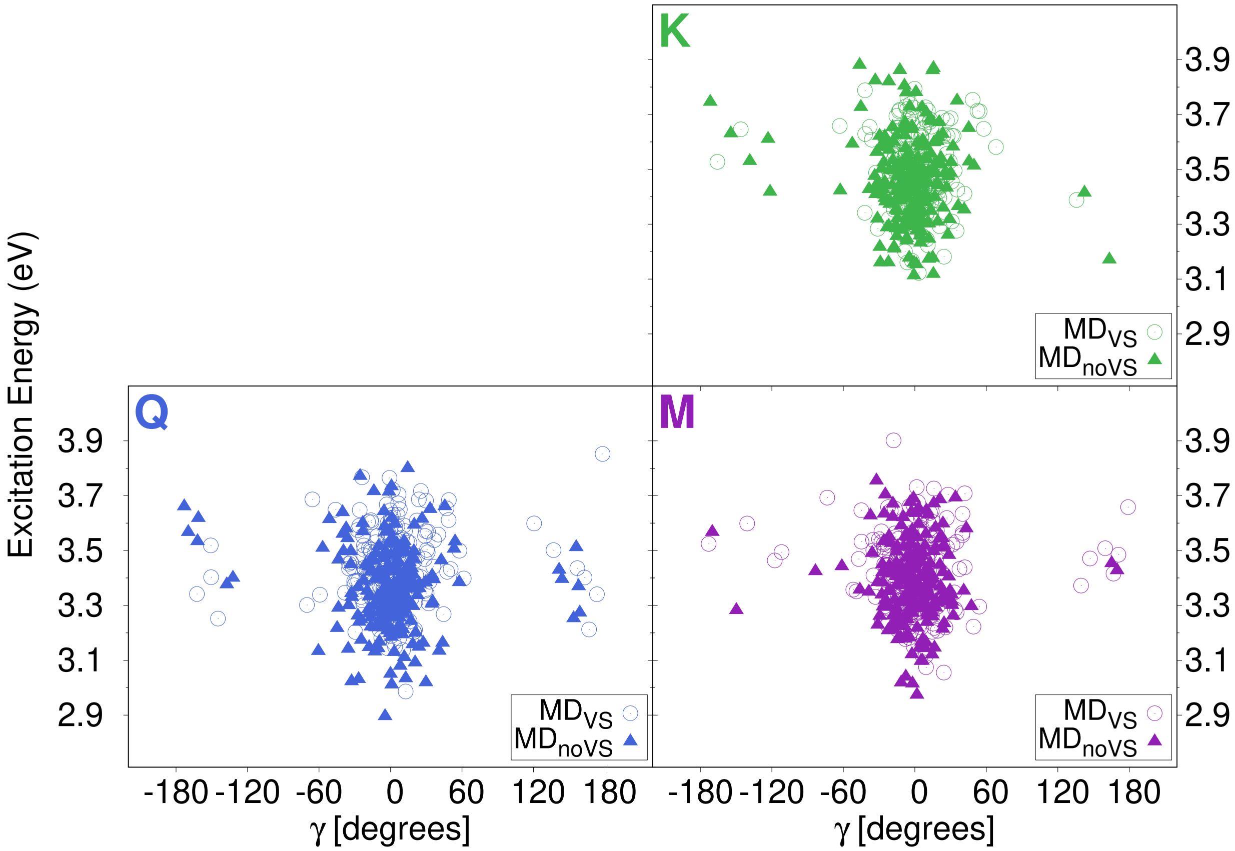


Figure S10: QM/FQ Excitation Energies of the first electronic transition as a function of $\gamma$ dihedral angle.
